# Increasing hospital administrative efficiency via optimized office automation systems: A PDCA cycle approach

**DOI:** 10.1371/journal.pone.0321475

**Published:** 2025-04-29

**Authors:** Ruiwen Lu, Yiming Zhang, Likang Luo, Quan Yuan, Shuihong Chen, Na Lv, Fenfang Zheng, Guofang Deng, Yuefeng Ma, Jie Xu, Zexin Chen, Fengjiang Zhang

**Affiliations:** 1 Office of Party and Government Affairs, The Second Affiliated Hospital, Zhejiang University School of Medicine, Hangzhou, Zhejiang, China; 2 Department of Artificial Intelligence and Information, The Second Affiliated Hospital, Zhejiang University School of Medicine, Hangzhou, Zhejiang, China; 3 The School of Management, Zhejiang University, Hangzhou, Zhejiang, China; 4 Department of Quality Management Office, The Second Affiliated Hospital, Zhejiang University School of Medicine, Hangzhou, Zhejiang, China; 5 Office of Scientific Research, The Second Affiliated Hospital, Zhejiang University School of Medicine, Hangzhou, Zhejiang, China; National center for chronic and non-communicable diesease prevention and control, CHINA

## Abstract

**Background:**

Increasing hospital administrative efficiency is crucial with regard to efforts to manage competitive pressures in healthcare. Despite extensive research on healthcare optimization, the role of Office Automation Systems (OASs) in administrative efficiency in the hospital context remains underexplored. This study addresses this gap by employing the plan–do–check–act (PDCA) cycle to improve administrative processes at the Second Affiliated Hospital, Zhejiang University School of Medicine.

**Methods:**

As part of this study, the root causes of inefficiencies in administrative processes were analyzed, and a PDCA cycle approach was implemented to address these inefficiencies. Monthly training sessions were conducted, and rankings were displayed to raise awareness of this topic among employees. A total of 41 high-frequency processes were monitored and divided into the categories of medical research, clinical use, and daily administrative work. Data were collected from 2021 to 2023, and statistical analyses were conducted via SPSS and R software.

**Results:**

Significant improvements in process implementation times were observed. With regard to clinical use processes, the median number of days required to process hospitalization and discharge procedures decreased from 4.33 in 2021 to 0.04 in 2023 (P < 0.001). Similarly, the number of days spent processing software requests decreased from 75.98 in 2021 to 31.33 in 2023 (P < 0.001). With respect to medical research processes, the median number of days required for animal laboratory access training applications decreased from 10.87 to 1.24 days (P < 0.01). Daily administrative processes also significantly improved, such that the median number of days spent on processing fixed asset disposal applications decreased from 5.96 to 4.21 (P < 0.01).

**Conclusion:**

The application of the PDCA cycle significantly improved the efficiency of hospital administrative processes, thus highlighting the potential of this approach to serve as a valuable tool for continuous efforts to improve hospital administration. This study provides a framework for other hospitals seeking to increase their administrative efficiency via digital transformation and continuous quality improvement.

## 1 Introduction

In response to the challenges associated with efforts to innovate and improve efficiency both at the policy level and in daily operations, many hospitals are undergoing notable changes [1]. Enhancing efficiency has become a critical strategy that hospitals can use to navigate the increasingly strong competitive pressures that characterize the healthcare market. [2]. Despite the proliferation of research on optimization in the field of healthcare, few studies have investigated the role played by the administrative office mode in efficient administration.

China is vigorously developing its “digital economy” and “low-carbon economy” [3]. Simultaneously, against the backdrop of vigorous efforts to promote digital reform in Zhejiang Province, the development of hospital office automation (OA) processes is also undergoing rapid advancement. In comparison with traditional manual operations, online process implementation makes administrative tasks more standardized, paperless, and cost-effective [4]. OA also promotes faster communication and circulation among internal departments within the hospital. Since the introduction of the North American Industry Classification System (NAICS) codes in 2017, online processes have been established to govern administrative transactions. However, as more processes are established, the time required to complete these processes begins to affect the overall efficiency of administrative work directly.

Previous studies have investigated how OA systems are applied in practice and how these systems significantly improve administrative efficiency [5]. However, only limited research has explored the use of OA systems and their impacts on administrative efficiency in hospital settings. The aim of this study is to fill this gap. Specifically, with regard to the use of OA systems, we focus on employees’ perceptions of organizational and work practices, processes, relationships, and cultures [6]. A preliminary investigation revealed that most processes involve many functional departments, such as the Scientific Research Office, Finance Office, Ethics Office, Department of Pharmacy, and Department of Medical Engineering. An OA system can standardize the specific management responsibilities of these departments. Each functional department establishes its own approval processes or develops a corresponding management platform that is maintained with the assistance of its own software in a manner that lacks information interoperability, thus leading to information silos, problems with repeated submissions and approvals, and increased burdens on employees.

Under these circumstances, we used various means to publicize, supervise and guide the rapid handling of each link in such a process and to improve employees’ awareness of the need to accomplish these tasks in a timely manner. However, we believe that quality improvement projects must be implemented, and continuous improvement must be achieved on the basis of the plan–do–check–act (PDCA) cycle. The PDCA cycle, which is also known as the Deming cycle, is a four-phase iterative model that has been widely used to promote continuous improvement in quality management, including in areas such as surgical procedures and standardized nursing care. Recent studies have highlighted the effectiveness with regard to efforts to enhance administrative processes [7,8]. The PDCA cycle is used to improve administrative efficiency and exhibits dynamic characteristics that can be adjusted on the basis of the results of the time required to implement the OA process. Furthermore, this model can account for other improvements, such as an increased protocol execution rate or adjustments to the approvers. Improving hospital administration via the implementation of the PDCA cycle thus represents a more comprehensive and continuous approach [9]. Studies have reported that the PDCA cycle management approach significantly reduces decision-to-delivery interval time and lowers the neonatal asphyxia rate while simultaneously ensuring favorable maternal outcomes [10]. However, the degree to which hospital clinical department managers, particularly department heads, understand the PDCA cycle is relatively limited [11].

Nevertheless, PDCA exhibits certain deficiencies in both theoretical and practical operational terms. As a result, we implemented the following innovative adjustments: (I) Cultivating flexible thinking: We offered comprehensive training programs to all personnel involved in the approval process with the goal of ensuring that they have a profound understanding of the fact that PDCA is a malleable framework rather than an inflexible dogma. In response to critical and urgent approval processes, immediate emergency handling measures (act) can be implemented. The iterative cycles of plan, do, and check can subsequently be performed in an orderly manner with the goal of swiftly resolving the issues at hand. (II) Diversifying data collection: We used multiple data collection approaches to ensure the precision and comprehensiveness of the data referenced in this research. By installing sensors on medical equipment to capture real-time data continuously, including data concerning temperature, pressure, and vibration, and integrating these data with the product quality data obtained via manual spot inspections, a more robust and reliable data foundation is constructed. This foundation facilitates the provision of substantial support with respect to approval processes pertaining to equipment repair and the acquisition of new equipment. (III) Optimizing resource investment: Before the PDCA cycle is implemented, on the basis of a meticulous consideration of various factors such as the influence of the problem on organizational objectives, the feasibility of resolving the problem, and the anticipated returns, we determine which problems deserve preferential allocation of resources devoted to improvement. Consequently, as part of this process optimization and improvement project, we initially focus on resolving the issue of a substantial backlog and the most time-consuming forms of expense reimbursement within this process. By focusing the limited available resources on these key areas, we enhance the efficiency of resource utilization. (Ⅳ) Strengthening the support and participation of senior management: By publicizing the processing time durations associated with various processes at regular hospital leadership meetings, we highlight successful cases and application achievements of the PDCA approach in the context of actual projects to senior managers. This approach effectively increases their attention to this topic and stimulates their active engagement. Only through such changes is it possible to maintain efficient and high-quality administration [9].

In light of the notable lack of standardized accelerated processing programs, this study emphasizes a project that focuses on time management challenges and the process of designing a process monitoring solution that can reduce waiting times while simultaneously maintaining current labor costs. The implementation of this improvement project can help accelerate processing time and improve administrative efficiency.

## 2 Methods

### 2.1 Workflow and design

A root cause analysis conducted by the project team revealed that employees lack of awareness of the need to implement such processes in a timely manner; another relevant issue in this context pertains to the establishment of process links. We plan to provide monthly training and display the ranks of various processing and signing times at the administrative office meeting with the goal of raising awareness of the need to implement processes in a timely manner among administrative personnel. Simultaneously, the process improvement working group aims to produce a thorough investigation of various functional departments with the aims of exploring plans for process improvement and addressing bottlenecks in relevant links that can affect processing speed. Therefore, the focus of this retrospective study is on the task of enhancing employees’ awareness of the need to implement processes in a timely manner as well as to improve various links within these processes with the aims of fundamentally improving those processes and decreasing the corresponding processing time.

### 2.2 Inclusion and exclusion criteria

The inclusion criterion for this research was as follows: individuals who served as hospital employees with responsibility for the implementation of administrative processes at the Second Affiliated Hospital, Zhejiang University School of Medicine between January 2021 and December 2023. The exclusion criterion was as follows: administrative processes that were either interrupted or canceled.

### 2.3 Participants

On the basis of discussions between the process improvement working group and each functional department, 41 widely used and high-frequency processes were selected for monitoring; all records generated by these processes in 2023 were included in the analysis. The sample selection process employed in this research was based on 41 core processes that were frequently included among the daily administrative process of the hospital. These processes covered three categories: medical research, clinical use and daily administrative work. We selected these processes on the basis of their repetition in the management context, their broad reach, and the significant impacts of the corresponding processing times on overall administrative efficiency. After the processes were divided into categories on the basis of the departments to which they belonged, 10 (24%) processes were found to be related to medical research, 15 (37%) were observed to pertain primarily to clinical use, and 16 (39%) were revealed to be related to the daily work of the administrative department. The data collection methods used in this context included system export and manual verification to ensure data accuracy and completeness. This study was reviewed and approved by the Human Research Ethics Committee of the Second Affiliated Hospital, Zhejiang University School of Medicine (Hangzhou, China).

### 2.4 Implementation of the PDCA cycle for office automation

1) Plan

Low implementation rate of the approval process: Accelerating the approval process can decrease the corresponding waiting time. However, it is necessary to ensure that the labor cost does not increase, which is the greatest challenge associated with this program. A relevant countermeasure in this context involved understanding the current difficulties on the basis of in-depth research and examining the cause of inefficiency in each case. On the basis of the research highlighted above, we collaborated with the IT Center to develop a process efficiency analysis, which included statistics concerning the average process link time, a ranking of individual average processing time, and a ranking of average processing time. Moreover, we established a quality management team. The members of this team were responsible for responding to daily questions, and the team leader was responsible for summarizing common questions and providing answers online. These team members also conducted one-on-one meetings with inefficient approvers, summarized common causes, and refrained from penalizing those individuals publicly to avoid eliciting any resentment.

2) **Do**

The quality management team collected data concerning the processing time associated with all administrative departments on a monthly basis, identified the causes of inefficiency, analyzed the reasons underlying each case of wasted time, formulated corresponding measures, and implemented those measures the following month. The quality improvement project started in January 2022. Monthly statistics were published in administrative office meetings, and the functional department director used these statistics to remind employees to implement processes promptly. Through meeting reminders, online training, and other methods, the training process for new online processes was strengthened, and the “ask at most one person” NAICS query function was developed to enable employees to understand the use of processes, select processes correctly and understand the process flow with the aim of preventing delays.

3) **Check**

(I) Monthly data summary and corresponding countermeasures were reported to the quality management team. (II) Weekly spot checks of measure implementation were conducted by the director and the team members. (III) For processes that featured particularly long average processing times, we calculated statistics concerning each link of the process and identified the links that mandated such long processing times.

4) Act

(I) Two editions of OA process-specific training programs were formulated and updated. (II) Multisectoral monitoring and coordination mechanisms were established. (III) Regular inspections were conducted, and an evaluation system was constructed.

### 2.5 Statistical analysis

All the statistical analyses were conducted with the assistance of SPSS software (version 29.0) and R software (version 4.2.2). For quantitative data, variables that exhibited a normal distribution are presented in terms of the means ± standard deviations (SDs); these variables were analyzed via independent sample t tests. Variables that did not exhibit a normal distribution are presented in terms of medians and interquartile ranges (IQRs); with regard to these variables, comparisons were made via the nonparametric Mann‒Whitney U test and the Kruskal‒Wallis test. Categorical data are presented in terms of numbers (%); these data were analyzed via the chi-square (χ2) test. A P value less than 0.05 was deemed to indicate statistical significance across all analyses.

## 3 Results

### 3.1 Results of the data collection process

**Fig 1:**
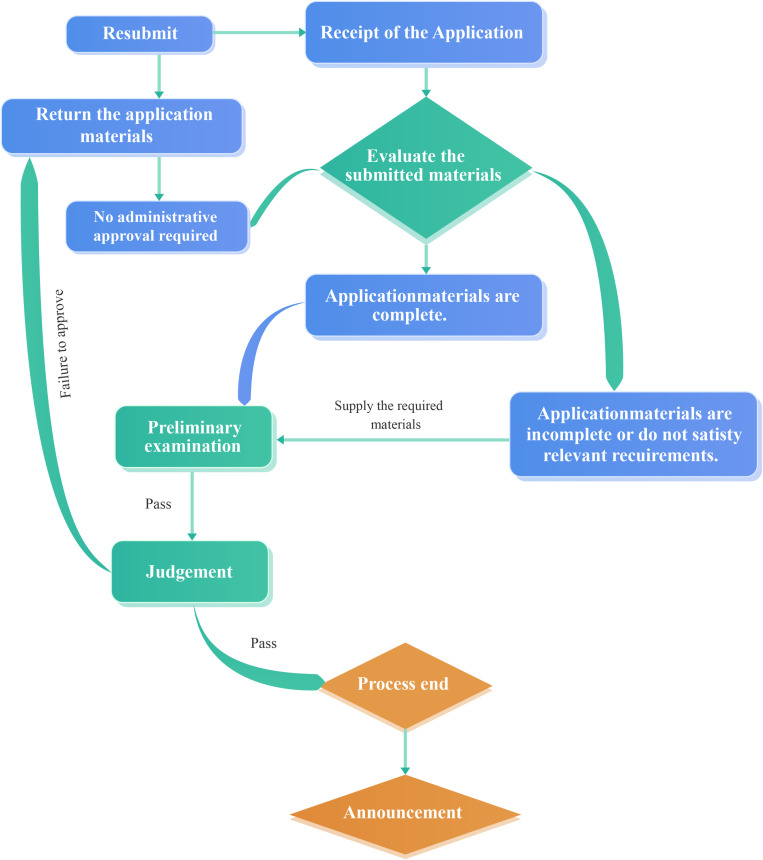
Administrative approval process chart before optimization.

**Fig 2:**
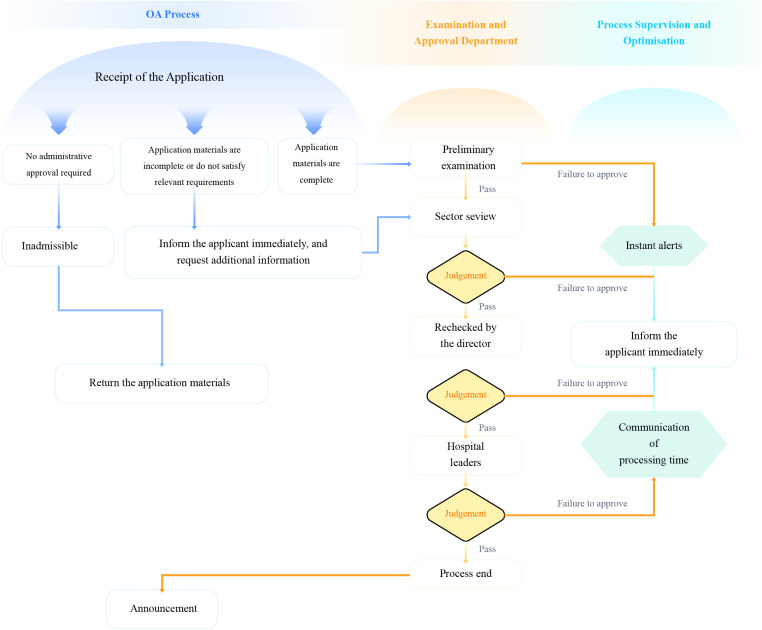
Administrative approval process optimization chart.

The research period, which extended from January 2021 to December 2023, was divided into three stages by year. We started our analysis by examining the descriptive statistics and correlation matrix, as presented in Table 1. After two years of refinement, the preset system can seamlessly assign departmental reviewers and relevant leaders to each specific approval category. Throughout this process, applicants can conveniently monitor the real-time status of their applications via their mobile phones or computers, thus ensuring transparency and traceability at every step of the process. In the event that any issues with the submitted materials arise, reviewers at each stage possess the authority to request revisions for materials that do not meet the necessary criteria. The administrative approval process chart before optimization and the optimization chart are presented in Figs 1 and 2. The conceptual design of the model includes details regarding the steps in the processes. For cases in which a process application becomes necessary, the model establishes a connection with the designated departments and approvers. Whenever an application appears to be missing material, the model reverts to the initial submission phase to determine the most appropriate available application program.

**Table 1:** Baseline information and characteristics of OA processes–Initial examination the relationships among the cross-sectoral approach, the participation of hospital leaders and efficiency.

id	Group	OA Process	Cross-sectoral	Participation of hospital leaders	P50		
					2021	2022	2023
1	Clinical work	Scoring of the Medical Record Examination	N	N	6.79	1.56	1.72
2		Temporary drug supply application	N	N	2.04	2.05	1.48
3		Clinical research center access training	N	N	5.03	4.64	3.03
4		Specialist (or specialized) outpatient clinic suspension application process	Y	N	0.1	0.01	0.01
5		Hospitalization and discharge procedure	Y	N	4.33	0.06	0.04
6		Outpatient scheduling application process	Y	N	0.05	0.04	0.12
7		Application process for medical consumables (temporary use)	Y	Y	3.98	4.12	4.03
8		Information system data collection application	Y	N	10.79	12.30	10.80
9		Approval process for revisions to hospitalized medical records	Y	N	1.00	1.05	2.15
10		Application process for modification of the “hospitalization type” of prehospitalized patients	Y	N	1.17	0.97	1.28
11		Application process for the suspension of specialist (or specialized) outpatient services	Y	N	0.46	0.49	0.53
12		Outpatient complaint application process	N	N	5.92	6.33	7.16
13		Requests for medical equipment and office equipment	Y	Y	6.85	7.67	5.98
14		Application for leaves of absence on the part of resident physicians	Y	N	1.61	1.85	2.39
15		Software requirements request	Y	N	75.98	56.28	31.33
16	Medical science research	Application procedure for the intrainstitutional transfer of research funds	N	N	4.71	3.94	3.71
17		Database application	Y	N	1.71	1.04	1.16
18		Animal laboratory access training application	N	N	10.87	7.14	1.24
19		Academic paper submission registration process	Y	N	4.85	4.35	3.73
20		Request for payment	Y	Y	10.95	5.79	6.75
21		Application for recharging clinical trial funding cards	Y	N	1.86	1.07	1.77
22		Reimbursement for research funding	Y	Y	32.81	16.38	28.94
23		Application procedure for the reimbursement of special funds (material and test processing fees)	Y	Y	38.68	29.21	40.73
24		Application procedure for the reimbursement of special funds (labor costs)	Y	Y	18.86	15.70	19.77
25		Reimbursement of page charges	Y	Y	25.59	14.64	23.98
26	Daily affairs of the administration	Registration form for matters discussed at hospital leadership conferences	N	N	5.96	5.64	4.21
27		Administrative general duty record process	N	N	0.052	0.079	0.032
28		Fixed asset disposal application process	Y	Y	7.62	5.77	4.94
29		Medical equipment maintenance and warranty application demonstration process	Y	Y	7.53	4.07	3.77
30		Access control authority application	Y	N	2.37	1.81	1.84
31		Approval of new and revised systems	Y	Y	18.85	14.01	17.73
32		Departmental fire self-inspection form	N	N	0.53	0.75	0.67
33		Motor vehicle parking application and monthly parking	N	N	0.41	0.47	0.39
34		Application for software privileges	Y	N	5.05	6.04	5.92
35		Outsourcing contract quality monitoring platform	Y	Y	5.91	4.70	5.01
36		Authorization to enter Zhejiang University	Y	N	0.80	1.23	1.77
37		The process by which the official seal of the hospital is stamped and the signatures of legal persons are provided	Y	N	0.79	0.74	0.93
38		Administrative issuance	Y	Y	3.76	2.98	5.19
39		Receiving and circulating documents	Y	Y	2.61	1.29	6.04
40		Online approval application for economic contracts	Y	Y	40.05	34.02	38.93
41		Computer equipment application form	Y	Y	9.89	5.03	5.88

### 3.2 PDCA can improve clinical use efficiency

**Fig 3:**
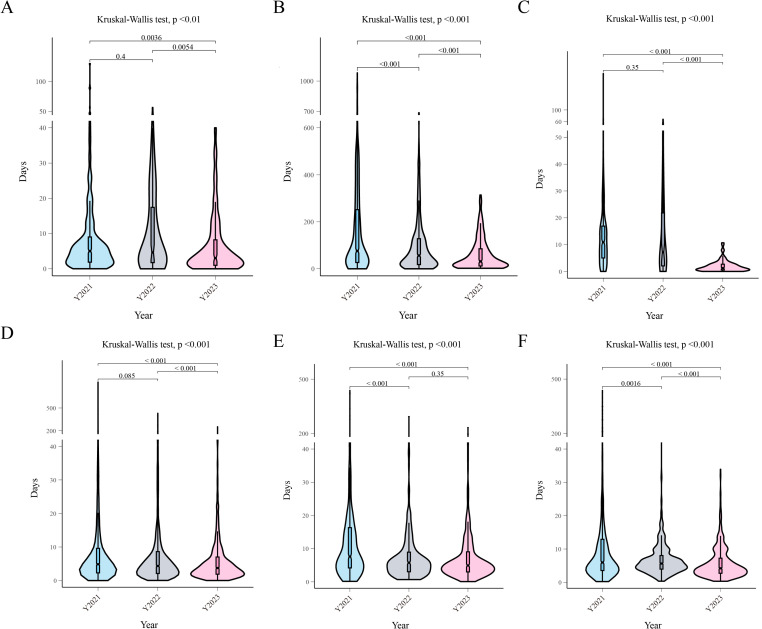
PDCA can improve clinical use, medical research, and daily administrative task efficiency. (A) Violin plot of acquisition days associated with the clinical research center access training process. (B) Violin plot of the software request requirement process. (C) Violin plot of the animal laboratory access training application process. (D) Violin plot of the academic paper submission registration process. (E) Violin plot of the fixed asset disposal application process. (F) Violin plot of the registration form process pertaining to matters discussed at the hospital leadership conference.

In our study, 15 of 41 processes were revealed to be related to clinical use. In light of our optimization process, we found that six of these processes exhibited significant improvement (Table 2). We observed that hospitalization and discharge procedures improved significantly. The median number of days spent on processing decreased from 4.33 in 2021 to 0.04 in 2023 (P value < 0.001). In addition, we found that the process of clinical research center access training improved from 2021–2023 (P value < 0.01, Fig 3A). A further analysis of the two groups revealed no significant difference between 2021 and 2022 in this regard (P value > 0.05). In parallel, we observed a notable improvement in the number of software requirement requests. In 2021, we received 1,055 process requests; in 2022, we received 542 requests; and in 2023, we received 235 requests. We observed that the median number of days spent on processing decreased from 75.98 to 31.33 (P value < 0.001, Fig 3B). Significantly, a pairwise comparison revealed that the efficiency of this process improves annually.

**Table 2:** The results of processes pertaining to clinical use.

Name	Total number	Days (medians and IQR)			P value	Trend
		2021	2022	2023		
Application for leaves of absence on the part of resident physicians	568	1.613(0.792–3.301)	1.851(0.893–3.736)	2.39(1.415–4.506)	<0.05	increase
Application Process for Medical Consumables (Temporary Use)	1389	3.984(2.477–6.465)	4.117(2.619–6.042)	4.025(2.125–7.027)	0.625	/
Application process for modification of the “hospitalization type” of prehospitalized patients	2502	1.166(0.272–3.715)	0.968(0.26–1.982)	1.283(0.374–3.092)	<0.05	undulate
Application process for the suspension of specialist (or specialized) outpatient services	7935	0.456(0.082–0.876)	0.486(0.106–0.861)	0.529(0.103–0.946)	<0.05	increase
Approval process for revisions to hospitalized medical records	1462	0.996(0.347–2.839)	1.053(0.695–2.954)	2.149(0.931–4.142)	<0.05	increase
Clinical research center access training	645	5.031(1.879–9.032)	4.643(1.75–17.433)	3.028(0.998–8.222)	<0.05	reduce
Hospitalization and discharge procedure	3075	4.325(0.115–40.088)	0.057(0.023–0.167)	0.041(0.017–0.108)	<0.05	reduce
Information system data collection application	845	10.794(3.917–34.123)	12.305(4.854–37.03)	10.803(4.994–24.822)	0.4166	/
Outpatient complaint application process	369	5.917(3.141–9.969)	6.327(3.297–9.512)	7.161(4.823–13.238)	<0.05	increase
Outpatient scheduling application process	5297	0.045(0.009–0.55)	0.042(0.006–0.512)	0.12(0.009–0.633)	<0.05	increase
Requests for medical equipment and office equipment	2326	6.852(3.029–16.94)	7.666(4.396–18.701)	5.981(3.707–13.258)	<0.05	undulate
Scoring of the Medical Record Examination	3906	6.787(1.976–12.049)	1.563(0.585–4.673)	1.724(0.737–3.389)	<0.05	reduce
Software requirements request	1832	75.981(26.819–251.542)	56.284(17.94–127.986)	31.332(12.074–85.23)	<0.05	reduce
Specialist (or specialized) outpatient clinic suspension application process	7935	0.456(0.082–0.876)	0.486(0.106–0.861)	0.529(0.103–0.946)	<0.05	increase
Temporary drug supply application	4736	2.039(0.987–3.886)	2.045(1.014–3.342)	1.477(0.899–2.937)	<0.05	reduce

### 3.3 PDCA can improve medical research efficiency

We observed that 10 of our 41 processes were related to medical science research. Three of these processes exhibited significant improvement, including an animal laboratory access training application process and an academic paper submission registration process (Table 3). With respect to the animal laboratory access training application, the median number of days spent on processing decreased from 10.87 to 1.24 (P value < 0.01, Fig 3C). In parallel, we observed a notable improvement in the academic paper submission registration process. The median number of days spent on processing decreased from 4.85 in 2021 to 3.73 in 2023 (P value < 0.01, Fig 3D). In both of these processes, pairwise comparisons revealed no statistically significant difference between 2021 and 2022 alongside a significant increase in 2023.

**Table 3:** The results of processes pertaining to medical research.

Name	Total number	Days (medians and IQR)			P value	Trend
		2021	2022	2023		
Academic paper submission registration process	3923	4.851(2.276–9.596)	4.349(2.097–8.636)	3.73(1.883–7.005)	<0.05	reduce
Animal laboratory access training application	411	10.87(5.038–16.891)	7.141(2.07–21.799)	1.241(0.559–2.696)	<0.05	reduce
Application for recharging clinical trial funding cards	730	1.859(0.932–3.667)	1.074(0.747–2.796)	1.773(0.92–3.711)	<0.05	undulate
Application procedure for the intrainstitutional transfer of research funds	803	4.708(2.746–6.636)	3.939(2.423–5.703)	3.714(2.051–5.481)	<0.05	reduce
Application procedure for the reimbursement of special funds (labor costs)	1153	18.863(13.761–26.949)	15.703(10.839–21.022)	19.768(13.813–26.444)	<0.05	undulate
Application procedure for the reimbursement of special funds (material and test processing fees)	3063	38.684(24.417–52.866)	29.214(19.764–46.911)	40.728(32.266–51.814)	<0.05	undulate
Database application	2518	1.706(0.883–3.777)	1.042(0.712–2.919)	1.164(0.793–2.775)	<0.05	reduce
Reimbursement of page charges	1444	25.593(19.25–36.619)	14.635(9.997–21.66)	23.979(17.954–32.475)	<0.05	undulate
Reimbursement of research funding	806	32.812(22.961–48.897)	16.379(10.676–25.751)	28.936(20.3–45.896)	<0.05	undulate
Request for payment	3131	10.954(5.365–21.785)	5.789(3.031–10.984)	6.75(3.761–12.673)	<0.05	undulate

### 3.4 PDCA can improve daily administrative task efficiency

A total of 16 of the 41 processes on which this research focused were related to daily administrative tasks (Table 4). Following our optimization, we found that four of these processes exhibited significant improvement, such as the processes pertaining to computer equipment applications, fixed asset disposal applications, medical equipment maintenance, and registration forms related to issues that were discussed at hospital leadership conferences. We found that the median number of days required by the fixed asset disposal application process decreased significantly (P value < 0.01, Fig 3E). However, no significant improvement was observed between 2022 and 2023 (P value > 0.05). In addition, the median number of days spent processing registration forms pertaining to matters discussed at hospital leadership conferences decreased from 5.96 to 4.21 (P value < 0.01, Fig 3F). Moreover, we found that the efficiency of this process gradually improved over time.

**Table 4:** The results of processes pertaining to daily affairs of the administration.

Name	Total number	Days (medians and IQR)			P value	Trend
		2021	2022	2023		
Access control authority application	10800	2.367(1.041–6.047)	1.806(0.9–5.005)	1.843(0.768–5.117)	<0.05	reduce
Administrative general duty record process	3190	0.052(0.004–1.181)	0.079(0.002–1.646)	0.032(0.001–0.743)	<0.05	undulate
Administrative issuance	583	3.76(1.848–5.993)	2.983(1.322–5.685)	5.19(2.981–6.996)	<0.05	undulate
Application for software privileges	910	5.045(1.25–16.677)	6.042(2.007–20.817)	5.919(2.725–17.41)	0.0649	/
Approval of new and revised systems	537	18.846(9.928–38.711)	14.015(10.087–23.911)	17.731(12.043–27.551)	0.0764	/
Computer equipment application form	743	9.887(4.853–26.018)	5.027(3.043–7.898)	5.885(3.091–12.856)	<0.05	reduce
Departmental fire self-inspection form	2385	0.534(0.051–2.461)	0.752(0.08–4.028)	0.675(0.084–2.658)	<0.05	undulate
Fixed asset disposal application process	1164	7.62(4.204–16.324)	5.775(3.044–8.945)	4.942(2.995–9.055)	<0.05	reduce
Medical equipment maintenance and warranty application demonstration process	667	7.529(3.672–20.053)	4.073(2.159–6.045)	3.772(1.799–6.156)	<0.05	reduce
Motor vehicle parking application and monthly parking	2504	0.414(0.028–1.095)	0.467(0.055–1.048)	0.387(0.04–0.93)	0.061	/
Online approval application for economic contracts	4115	40.051(23.914–63.83)	34.016(20.564–49.265)	38.928(23.74–60.942)	<0.05	undulate
Outsourcing contract quality monitoring platform	864	5.906(2.955–7.852)	4.697(3.629–6.773)	5.012(4.03–8.219)	<0.05	undulate
Receiving and circulating documents	5283	2.612(0.973–5.101)	1.29(0.742–3.631)	6.039(1.868–22.046)	<0.05	increase
Registration form for matters discussed at hospital leadership conferences	2385	5.964(3.537–12.906)	5.645(3.958–8.03)	4.211(2.718–7.252)	<0.05	reduce
The process by which the official seal of the hospital is stamped and the signatures of legal persons are provided	9436	0.79(0.196–2)	0.744(0.163–1.601)	0.927(0.286–2.257)	<0.05	increase
Zhejiang University pass (blue code) application process	2153	0.8(0.267–1.837)	1.23(0.785–2.726)	1.765(0.857–3.407)	<0.05	increase

## 4 Discussion

Administrative management is a crucial component of internal operations in hospitals. Hospitals should aim to promote professional and efficient administrative management by implementing reforms and adjustments in administrative work. By improving hospital administrative management systems, enhancing the professional skills of administrative staff, and strengthening hospitals’ administrative information management capabilities, hospitals can lay a solid foundation for the development of the medical and healthcare services that they provide. Effective hospital administration plays a crucial role in efforts to improve patient outcomes and overall healthcare efficiency [12]. The implementation of the PDCA cycle can also increase the efficiency of hospital administration via office automation. Research has revealed that integrating PDCA tools into internal hospital management significantly improves efficiency, fosters teamwork, and strengthens medical staff’s sense of self-identity [13–15].

The PDCA cycle is a continuous improvement methodology that has been widely used in various industries, including healthcare [16–21]. For example, the PDCA cycle is commonly used to manage nursing quality and oversee risk control in digestive endoscopy rooms [16]. One study that explored the cognition and application of the PDCA cycle among clinical department managers reported that most managers were aware of the PDCA cycle but did not always apply all the steps included in that cycle effectively. This study highlighted the importance of continuous training and support for managers that car allow them to take full advantage of the PDCA cycle to promote quality improvement in clinical settings [11]. A study in which the PDCA cycle was applied to standardized nursing management in the context of sepsis care revealed notable enhancements in both the compliance and completion rates of sepsis bundle treatments. This study emphasized the roles of continuous training and supervision in the process of enhancing the effectiveness of sepsis management protocols [22]. The implementation of the PDCA cycle in the context of nursing also effectively enhances the quality of care and medical outcomes for hemodialysis patients with internal arteriovenous fistulas, increases patient satisfaction, and decreases the likelihood of complications [23]. These studies have indicated that the PDCA cycle has been used in hospital management and clinical practice in various ways, ranging from efforts to improve management practices to attempts to enhance clinical protocols and patient safety. Our study is in line with the literature on the effectiveness of the PDCA cycle in healthcare settings. Another study revealed that the implementation of the PDCA cycle can improve clinical processes and patient care outcomes [24]. Our study extends these findings to the administrative domain by demonstrating that similar benefits can be achieved in nonclinical processes. The significant improvements observed in medical research processes, such as the reduction in the number of days spent on processing animal laboratory access training applications (i.e., from 10.87 to 1.24, P < 0.01), validate the versatility of the PDCA cycle.

This study employs an innovative approach by focusing on ways of optimizing and enhancing online processes. In the present social context, which is highly digitalized, this research capitalizes on the distinctive benefits of online approval. These benefits include the convenience of mobile approvals, intelligent recommendation and reminder features, innovative parallel approval mechanisms, visual process tracking and feedback capabilities, and the use of big data analysis to optimize decisions. The objective of this approach is to make the greatest enhancements with regard to administrative efficiency at minimal cost. It thus provides a framework that can be used by other hospitals to increase their administrative efficiency via digital transformation and continuous quality improvement.

Our findings indicate that the PDCA cycle can effectively address inefficiencies in administrative processes. For example, the median number of days spent on processing hospitalization and discharge procedures decreased from 4.33 in 2021 to 0.04 in 2023. This substantial reduction highlights the ability of the cycle to streamline complex administrative tasks, thereby ensuring quicker patient turnover and more efficient use of hospital resources. Similarly, the reduction in the number of days spent processing software requirement requests, i.e., from 75.98 to 31.33, highlights the efficacy of the PDCA cycle with regard to IT-related administrative tasks, which are crucial with respect to maintain and upgrade hospital systems.

The ‘do’ phase focuses on the implementation of targeted interventions on the basis of the identified inefficiencies. Monthly data collection and analysis enabled the quality management team to identify specific areas that required improvement [25]. Intervention approaches included optimizing process links, enhancing staff training, and utilizing digital tools to streamline operations. For example, the number of days spent processing software requirement requests decreased significantly from 75.98 in 2021 to 31.33 in 2023 (P < 0.001) as a result of these targeted improvements.

The ‘check’ phase involves the continuous monitoring of process performance and gathering feedback. Regular reporting and spot checks ensure that the interventions were implemented effectively and that any emerging issues were addressed promptly addressed. This phase was crucial with regard to efforts to maintain the momentum of improvements and ensure that these changes led to sustainable efficiency gains. The ‘act’ phase allows adaptive adjustments to be made on the basis of the performance data. By updating training programs and establishing multisectoral monitoring mechanisms, the hospital can continuously refine its processes. The improvement in the median number of days spent processing fixed asset disposal applications, i.e., from 5.96 to 4.21 (P < 0.01), exemplifies the dynamic nature of the PDCA cycle as a driver of ongoing improvements.

However, the opposite trend was observed with respect to some processes, such as the approval process associated with revisions to hospitalized medical records in the clinical use field. In light of the importance of medical quality and safety, the requirements for revisions to hospitalized medical records are more stringent. Thus, on the basis of departmental self-approval, the centralized approval process of the medical department was added, thus increasing processing time. A similar trend was observed in the Zhejiang University pass (blue code) application process, which is a routine administrative task that is driven by a change in the approval authority. The final reviewer in this process was changed from the director of the office to the chief of the personnel section, and the requirements of this process were changed, particularly by increasing the number of materials to be submitted and slowing the speed of approval. Approval times also increased in the context of application procedures pertaining to special fund reimbursement (Material and Test Processing Fees). With the addition of three new hospital districts in 2023 (as compared with the number of hospitals in 2021), the special fund program of hospitals grew rapidly as new hospital districts continued to be implemented, and the workload of finance and purchasing staff was very high. However, the number of staff did not increase in proportion to the increase in staffing levels; thus, it was not possible to achieve high efficiency with respect to the original reimbursement process.

In our study, we also address the issue of environmental changes and their impacts on the survey results. For example, confounding variables such as changes in hospital policy or staffing can impact the efficiency of the corresponding processes. However, the processes on which we focused avoided the influence of relevant confounding factors to the greatest extent possible and considered only the role of PDCA in the process of improving the whole process. One primary limitation in this regard pertains to the relatively small sample size of this research, as this study was confined to a single hospital setting, thus limiting the generalizability of the findings of this research to other hospitals or healthcare settings that are characterized by different administrative structures or levels of resources. Future studies with larger and more diverse samples are necessary to validate these findings across different contexts. This study may also be subject to various biases. For example, selection bias could occur if the processes chosen for monitoring were not representative of all administrative tasks. Additionally, performance bias may be a relevant issue if the staff are aware of the monitoring and adjust their behavior accordingly. These biases can affect the validity of the study results. Another limitation of this research lies in the potential variability in the fidelity of the implementation of the PDCA cycle. Inconsistent application of the principles of this cycle can lead to variable outcomes. This research should also include more relevant results, such as satisfaction, which can illustrate the effects of PDCA in several ways.

The PDCA cycle might drive changes or improvements in the OA system via 3 potential mechanisms. (I) Automation of data entry and processing: Through the use of technologies such as optical character recognition (OCR) and robotic process automation (RPA), the automation of data entry and straightforward data processing can be accomplished. For example, during the financial approval process, when applicants upload a substantial quantity of paper invoices to the OA system, OCR technology can automatically extract the textual information from the invoices and transform it into electronic data. (II) Intelligent decision support for efforts to optimize workflow decisions: For example, in the process of maintaining medical equipment maintenance and procuring new equipment, by installing sensors on medical equipment to collect operational data from such equipment (such as temperature, pressure, or vibration), the hospital can prearrange equipment maintenance schedules, optimize production plans, and import them into the OA process, thus mitigating any adverse effects of the untimely maintenance or acquisition of medical equipment on the hospital’s operational efficiency and medical quality. (III) Precise real-time monitoring of process progress: The status of each approval stage within the OA process can be presented in real time via the digital platform. Supervisors can inspect the progress of process implementation at any moment via a visual dashboard, promptly detect stages that represent bottlenecks in the process, and allocate resources to expedite the review.

Future research should focus on larger, multisite studies with the aim of increasing the generalizability of these findings. The inclusion of a diverse range of hospitals of varying sizes, locations, and administrative structures would provide a more comprehensive understanding of the effectiveness of the PDCA cycle across different settings. In addition, other types of PDCA, such as the integrated FOCUS-PDCA procedure (i.e., find, organize, clarify, understand, select-plan, do, check, act), can be considered and applied [26]. Moreover, the goals in this context set should be in alignment with the hospital’s overarching development strategy and vision and feature a well-defined time frame. Such a foundation can increase the team’s sense of urgency and implementation capabilities. Moreover, it is essential to guarantee that all departments can participate in the goal-setting process. As these departments have responsibility for task execution, they possess a more immediate awareness of the actual circumstances and latent issues associated with the work and can thus provide practical suggestions concerning equipment optimization, work process streamlining, etc. Longitudinal studies are also needed to assess the long-term sustainability of the improvements that can be achieved through the PDCA cycle. Exploring the integration of advanced tools, including artificial intelligence and machine learning innovations, with the PDCA cycle could increase process efficiency and effectiveness to an even higher level.

In conclusion, the optimization of the online approval process has profound and far-reaching implications in both the theoretical and practical domains. Theoretically, this approach can promote the modern advancement of management theories and refine the theoretical framework of information management. The optimization of online process approvals epitomizes the profound combination of information technology with management theories. From the perspective of organizational behavior theory, the optimization of online process approvals has transformed the work behavior patterns of relevant actors such as administrative personnel and approvers. In the domain of information management, the optimization of online process approvals offers novel theoretical requisites and practical trajectories pertaining to the collection, processing, storage, and utilization of information. Concurrently, the optimization of online process approvals involves significant theoretical concerns such as information security and privacy protection. As the approval process becomes increasingly digitized, ensuring the security of sensitive information, including medical and financial data, during the approval process has become highly important.

In terms of practical ramifications, our results reveal that the optimization of online process approvals has substantially curtailed the approval cycle, thus allowing the hospital to allocate administrative resources more precisely. Simultaneously, the optimization of online process approvals has provided approvers with more exhaustive and accurate information, thereby augmenting the scientific nature of decision-making. By formalizing the approval process and the corresponding standards, all approval steps are distinctly recorded and traceable, thus rendering the process standardized and transparent. Eventually, owing to accelerated pace of approval and the corresponding improvements to management efficiency, the hospital can address the needs of patients more promptly and mitigate the need for patient services. Such an efficient approval process empowers the hospital to perform more proficiently in terms of overall operational efficiency, service quality, etc., thereby enhancing the hospital’s brand image and promoting its comprehensive competitiveness.
